# A Smartphone Food Record App Developed for the Dutch National Food Consumption Survey: Relative Validity Study

**DOI:** 10.2196/50196

**Published:** 2024-02-09

**Authors:** Marga Ocké, Ceciel Simone Dinnissen, Coline van den Bogaard, Marja Beukers, José Drijvers, Eline Sanderman-Nawijn, Caroline van Rossum, Ido Toxopeus

**Affiliations:** 1 National Institute for Public Health and the Environment Bilthoven Netherlands

**Keywords:** relative validity, smartphone food record, 24-hour dietary recall, mobile app, national food consumption surveys, smartphone, food, food consumption, app, diet, dietary intake, nutrients, survey, mobile phone

## Abstract

**Background:**

In the Dutch National Food Consumption Survey, dietary intake has been assessed since 2003 through 24-hour dietary recalls using the GloboDiet software. A new self-administered smartphone food record app called DitEetIk! was developed for potential use in future surveys.

**Objective:**

This study aims to evaluate the data collected using the DitEetIk! app and its relative validity for food group, energy, and nutrient intake compared with the previous dietary assessment method (GloboDiet 24-hour dietary recalls).

**Methods:**

A total of 300 participants aged 18 to 79 years were recruited from a consumer panel. Participants were asked to keep a record of their consumption using the DitEetIk! app on 3 nonconsecutive days. Trained dietitians conducted a 24-hour dietary recall interview by telephone using the GloboDiet software (International Agency for Research on Cancer) regarding 1 of 3 DitEetIk! recording days. Nutrient intake was calculated using the NEVO database (version 2021/7.0). Relative validity was studied by comparing data from GloboDiet 24-hour dietary recalls and the DitEetIk app for the same day. Participants with implausible records, defined as days with energy intake of <0.6 or >3.0 basal metabolic rate, were excluded from the analyses. For 19 food groups and 29 nutrients, differences in median intake were assessed using the Wilcoxon signed rank test, and Spearman correlation coefficients were calculated. Bland-Altman plots with mean differences and 95% limits of agreement were created for energy intake and the contribution to energy intake from fat, carbohydrates, and protein.

**Results:**

A total of 227 participants completed a combination of a DitEetIk! app recording day and a 24-hour dietary recall interview for the same day. Of this group, 211 participants (n=104, 49.3% men and n=107, 50.7% women) had plausible recording days. Of all recorded food items, 12.8% (114/894) were entered via food barcode scanning, and 18.9% (169/894) were searched at the brand level. For 31% (5/16) of the food groups, the median intake assessed using the DitEetIk! app was >10% lower than that assessed using 24-hour dietary recalls; this was the case for fruit (*P*=.005), added fats (*P*=.001), milk and milk products (*P*=.02), cereal products (*P*=.01), and sauces (*P*<.001). This was also the case for 14% (4/29) of the nutrients (all *P*<.001). Regarding mean intake, differences were generally smaller. Regarding energy intake, the mean difference and 95% limits of agreement were 14 kcal (−1096 to 1124). Spearman correlation coefficients between intake assessed using the DitEetIk! app and 24-hour dietary recalls ranged from 0.48 to 0.88 (median 0.78) for food groups and from 0.58 to 0.90 (median 0.72) for nutrients.

**Conclusions:**

Compared with GloboDiet 24-hour dietary recalls, the DitEetIk! app assessed similar mean energy intake levels but somewhat lower median intake levels for several food groups and nutrients.

## Introduction

### Background

Many countries conduct national food consumption surveys as these are considered important instruments for prioritizing, developing, and evaluating food policies [1,2]. Food consumption survey data can be used to assess adherence to food-based dietary guidelines and obtain insight into the food consumption patterns of a population. After combination with other data sources such as food composition databases, food contamination occurrence data, and life cycle assessment data, the nutritional adequacy of the diet, dietary exposure assessment to potentially hazardous substances, and the environmental impact of dietary patterns can be assessed [3-5].

The use of national food consumption survey data for multiple purposes requires dietary assessment methods that allow all consumed food items to be reported with detailed characterizations. On the basis of European projects such as the European Food Consumption Survey Method [6] and European Food Consumption Validation [7], the 2014 guidance on the European Union Menu methodology by the European Food Safety Authority prescribes the use of food records for children and 24-hour dietary recalls for adults as dietary assessment methods in European national food consumption surveys [8]. It is advised that trained personnel is employed to administer the recall interviews or conduct a food record completion interview with the participants [8,9]. The requirements of trained personnel and detailed food descriptions, the large number of and continuously changing food items on the market, and the need to handle all possible reported food items make food consumption surveys costly [10]. Moreover, these requirements also pose a burden on the survey participants. It has been suggested that this burden has led to declining and possibly selective participation rates in national food consumption surveys [1].

In the past decades, various tools for self-administered 24-hour dietary recalls or food records have become available [11]. These digital tools have the potential to be less resource intensive. Many users prefer these applications over the traditional methods as they can be used where and when it is convenient [12]. Information and communications technology–based applications also enable the use of user-friendly support functionalities that were not feasible with interviewer-based methods. For example, food package barcode scanning using the camera function of a smartphone [11] combined with a comprehensive branded food database reduces the time burden of searching for a product through a long list of food items. However, without the help of a trained interviewer, it might be challenging for participants to report all food items consumed and describe and quantify them accurately. These developments warrant further exploration of whether interviewer-based dietary assessments in national food consumption surveys can be replaced with self-administered dietary assessments using digital food record applications.

In the Dutch National Food Consumption Survey (DNFCS), dietary intake has been assessed through 24-hour dietary recalls by trained interviewers using the GloboDiet software since 2003 [13-16]. A new food record app for dietary assessment called DitEetIk!, which uses self-administration, was developed for potential use in future national dietary surveys in the Netherlands. A smartphone food record was chosen over a self-administered digital 24-hour dietary recall because of the availability of a branded food database in the Netherlands [17]. Such a database allows for specific food identification and can be used most optimally when keeping a food diary throughout the day on a mobile phone with a camera function for barcode scanning. Moreover, the level of smartphone ownership and use in the Netherlands is high. In 2019, smartphones were present in 89% of Dutch households [18].

### Objectives

To assess the suitability of the DitEetIk! app for future surveys, it is important to evaluate its quality and comparative validity against the method currently used in the DNFCS. Therefore, the aim of this study was to evaluate the level of detail regarding the food description obtained in the reported consumption in the DitEetIk! app and determine how well the DitEetIk! app is able to assess the daily intake of food groups, energy, and nutrients in comparison with dietitian-administered 24-hour dietary recalls using the GloboDiet software (International Agency for Research on Cancer) in adults. This study focused on systematic differences at the food group and nutrient levels, with random error being of secondary interest. To study the potential effects of the study design on the 24-hour dietary recall data, we also compared the GloboDiet 24-hour dietary recall data in this study with those of a matched population of the DNFCS 2019 to 2021 [19].

## Methods

### Recruitment

The intention was to collect data from 200 participants with sufficient variation in gender, age group, and educational level. To account for potential dropouts and invalid food recording data, 300 participants were recruited. Participants from a consumer panel of Kantar Netherlands were invited via email to take part in the study. Information regarding the privacy policy of the DitEetIk! app was provided. The sociodemographic characteristics of the panel members were known. Potential participants were eligible if they were aged between 18 and 79 years; were not institutionalized; did not participate in the DNFCS 2019 to 2021 [19] or the Eetmeter study [20]; did not use tube or parental feeding; and were able to use the DitEetIk! app on their smartphone, which had to run on the Android operating system version 7 or higher.

### Ethical Considerations

The Medical Research Ethics Committee of Utrecht University evaluated that the study was not subject to the Medical Research Involving Human Subjects Act of the Netherlands (dossier 21/686). All study participants provided written informed consent. After completion of the study, participants received an incentive bonus (NIPOints to be exchanged for a gift card or coupon).

### DitEetIk! App

#### Objective of the DitEetIk! App

In the Dutch language, “Dit eet ik” means “This is what I eat.” The DitEetIk! app was developed specifically for the objectives of the DNFCS. The food description had to be specific enough to provide insights into the intake of nutrients, the exposure to chemicals relevant from a food safety point of view, and the environmental impact of the diet of the Dutch general population aged 1 to 79 years.

#### Development Process

The DitEetIk! app was developed using an agile approach. Various usability tests and focus group sessions were part of this process. The development period lasted approximately 3 years, with a team of app builders, data managers, dietitians, and nutritionists. Technical development was conducted by Dienst ICT Uitvoering in collaboration with National Institute for Public Health and the Environment for functional development and formative evaluation. Both are Dutch governmental organizations. Safety and General Data Protection Regulation issues were considered in the app development. The DitEetIk! app does not collect information that makes the participant identifiable. The user interface of the app is in Dutch (B1 level).

#### App Availability, Registration, and Instruction

Version 1.0 of the DitEetIk! app was developed for Android smartphones and is available on the Google Play store. Using the DitEetIk! app, a person can keep a food record for specific days upon invitation (ie, the DitEetIk! app can be used only after entering a participant number with matching gender and age). An instruction movie can be viewed at any time after registration. Moreover, to support participants, context-specific information and relevant sections of the instruction movie are available on each screen. Participants were instructed to record all food and drinks consumed from getting up one day to getting up the next day.

#### Push Notifications and Feedback

At several moments—before, during, and after the specified day—push notification messages are sent via the DitEetIk! app to the participants to help remind them of food recording and submission of the food record. The DitEetIk! app does not provide instant feedback to the participants regarding their food consumption as, for dietary monitoring, it is important not to influence the participants.

#### Main Menu

**Figure 1:**
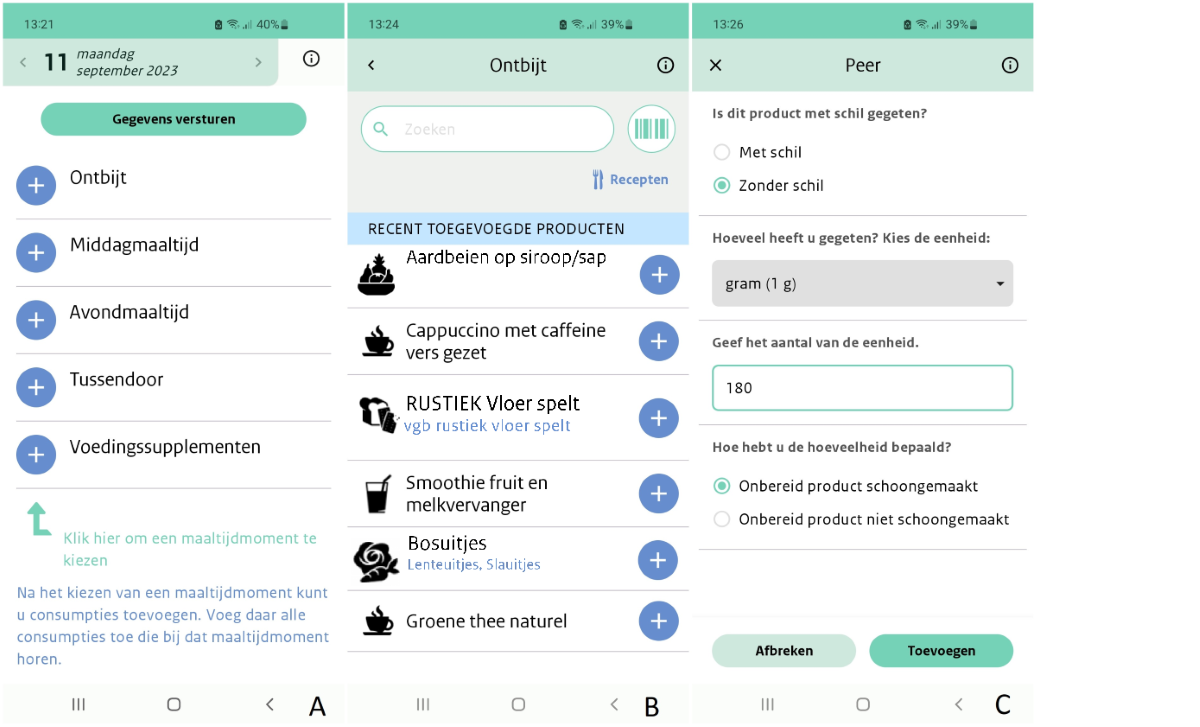
Screenshots of the DitEetIk! app depicting (A) eating occasions, (B) recently added products, and (C) food-specific follow-up questions.

Food recording is performed via a main menu where 4 eating occasions can be selected (ie, breakfast, midday meal, evening meal, and in between meals). If applicable, dietary supplements can be filled out separately (Figure 1A). After choosing breakfast, midday meal, or evening meal as eating occasions, the time and place of consumption need to be registered; for the occasion “in between meals,” time and place of consumption (eg, home or restaurant) are asked every time a food item is selected, whereas for dietary supplements, information regarding time and place of consumption is not asked for.

#### Food Recording

The DitEetIk! app food list in this study included 140,781 food items, of which 3432 (2.44%) were generic and the rest were a selection of branded food items from the Dutch-branded food database Dutch-branded food database (Levensmiddelendatabank [LEDA]; download date: October 27, 2021) [17]. The selection consisted of 70.89% (137,349/193,742) of the branded food items in the LEDA database that were matched to a generic product. Generic food items can be found through text searching. Branded food items can be found by scanning the barcode or, for predefined food groups, by selecting the brand and specific branded product once a generic food item is chosen. If the scanned food items are not included in the LEDA database or are not matched to generic products, they are not recognized in the app, and the participant has to record their food via text search. Once a product is selected and added to an eating occasion, the food item can also be found via “recently added products” and can be selected again (Figure 1B). A recipe feature is available it allows participants to create mixed dishes indicating quantities of foods that were used for the prepared dish as a whole or save frequently consumed combinations of foods. Commonly used mixed dishes are also available as prepared generic food items.

#### Follow-Up Questions for Each Food Item

After a food item is chosen or scanned, food-specific follow-up questions are asked regarding the preparation method, consumption with or without skin or peel, and quantity consumed (Figure 1C). In case a preparation method with fat (eg, frying or deep-frying) is chosen, the participant is asked to specify the type of fat used. The follow-up questions are defined at the generic food item level. For this reason, each branded food item is linked to a comparable generic food item. Consumed amounts can be indicated via various options that always include the weight in grams or volume in milliliters and often the number or fraction of household measures, natural units, or commercial units. For user-defined recipes, the fraction of the total recipe can be indicated as the consumed portion.

#### Data Submission and Output

When all consumptions for a day have been recorded, the participant can submit the food record. Upon submission, questions regarding completeness are asked, as well as whether the day was special or not regarding consumption. The data collected using the DitEetIk! app can be downloaded as CSV files that include information on eating occasions with time, place, and registered food items and the answers to the follow-up questions for each consumption.

### Study Design and Data Collection

Data were collected in the spring of 2022. Participants were asked to record all food, drinks, and dietary supplements consumed on 3 nonconsecutive days on the DitEetIk! app. The days were assigned by the DitEetIk! app in such a way that all days of the week were covered proportionally at the group level and that there were at least 7 days between each recording day. The day before each registration day, the participants received a push notification message on their smartphones. At the individual level, any combination of days could occur. In case the participant indicated that the day was not convenient for recording or did not record any food items, a replacement recording day was assigned automatically. This could be done maximally 3 times; thereafter, no new days were assigned, and the participant was excluded from further participation.

After 1 of the 3 DitEetIk! app recording days, participants were contacted by a trained dietitian for a 24-hour dietary recall interview by telephone. For the 24-hour dietary recall interviews, it was allowed to make an appointment with the participant; for practical reasons, they were not unannounced. The 24-hour dietary recalls were administered using the Dutch version of the GloboDiet software (version 2021-09-24). This software, which was previously called EPIC-Soft, has been described in detail elsewhere [16]. Briefly, the interview started by composing a quick list in which the participant was asked to roughly list all consumed food and drinks for 7 potential eating occasions with time and place of consumption. Food items were recalled starting from getting up in the morning until getting up the following day. In the second step, the interviewer specified each food item on the quick list using a series of follow-up questions applicable to that food item, for example, asking about preparation methods and, if relevant, the type of fat used. Mixed dishes could be entered as new individual recipes or as (adjustments of) standard recipes, which the software disaggregated into ingredients. Dietary supplements were explicitly asked about. Consumed amounts of the food items could be quantified in several ways: by means of quantities as shown in photos in a picture booklet with a series of 61 food photographs. or in household measures, units, and standard portions; by weight or volume; and by the proportion of a total recipe. Bread shapes were used to estimate the quantity of spreads. At various points, quality control of the data was incorporated into the GloboDiet software, for example, checks on missing quantities, probing questions on often forgotten food items, and checks on total intake of energy and macronutrients.

After the 3 food records were completed, the perceived usability of the DitEetIk! app was evaluated by the participants through a web-based questionnaire, the System Usability Scale (SUS) [21]. This is a widely used questionnaire for the evaluation of electronic devices and systems, including smartphone apps. It consists of 10 statements with response options on a 5-point Likert scale ranging from *strongly disagree* (1) to *strongly agree* (5). There are 5 positive statements alternated with 5 negative statements. The originally English-worded items were translated into Dutch, and the word “system” was replaced with “application” to make the questions more specific to the device.

### Data Handling

For the DitEetIk! app data, intake per food item per day was calculated by multiplying the weight of the chosen portion or serving by the number of portions. If applicable, food density (in the case of estimates in household measures or milliliters), an edible fraction (in the case of the inedible part), weight change because of food preparation (amount estimated as unprepared food), and percentage of fat absorption were applied to the calculation of the amount of food in grams per day in its consumed state. Similar calculations were performed using the GloboDiet software for the 24-hour dietary recalls [16]. All food items reported in the GloboDiet 24-hour dietary recalls and the DitEetIk! app were categorized into the food groups mentioned in the Wheel of Five Dutch food-based dietary guidelines [22,23]. Subsequently, consumption of food groups per person per day was calculated.

For both GloboDiet and DitEetIk! data, intake of energy and nutrients per person per day was calculated by multiplying the consumed amount of food by the nutrient level per gram of food and adding the nutrient intake of all food items consumed in a day. Information on food composition was obtained from the NEVO database (version 2021/7.0) [24]. Dietary supplements were not considered in the calculation of nutrient intake.

All extremely high values in energy, nutrient, and food group intake in the 24-hour dietary recalls were evaluated using the same methodology as in the DNFCS [15]. The food items that contributed the most to the high intake values were checked for obvious errors. None of the extreme intake values were judged as unlikely.

The equations developed by Henry [25] were applied to calculate the estimated basal metabolic rate (BMR) using height and weight information provided by the participants in the DitEetIk! app. The average ratio of energy intake to BMR was calculated, as well as the percentage of extreme energy reporters (ie, those participants with a ratio of <0.6 or >3.0). Any day with such an implausible extreme of energy intake in the DitEetIk! app was excluded from further analyses.

### Assessment of the Effect of Study Design on the GloboDiet 24-Hour Dietary Recall Results

The GloboDiet 24-hour dietary recall interview was always conducted after the recording in the DitEetIk! app. This might have influenced the results because of potential memory or learning effects [26]. The extent to which this occurred was estimated by comparing the results of the GloboDiet 24-hour dietary recalls in this study with the findings among participants in the DNFCS 2019 to 2021, in which 24-hour dietary recalls were collected using the same software. We only used the first GloboDiet 24-hour dietary recall interviews of these participants. Each participant in the DitEetIk! app evaluation study was matched with a participant in the DNFCS 2019 to 2021 based on characteristics associated with dietary intake, that is, age (5-year classes), gender, weight (10-kg classes), and educational level (3 classes). This provided matches for 86.3% (182/211) of the participants. For those without a match, the matching characteristics were relaxed, starting with educational level (28 matches) followed by age (1 match). For participants in the evaluation study with multiple possible matches in the DNFCS 2019 to 2021, a person with the same or the closest height was chosen (182 matches).

### Statistical Analysis

For the analyses, only DitEetIk! data for the day that was recalled using the GloboDiet software were used. Frequency analyses were conducted to describe the population of DitEetIk! app users and the matched participants of the DNFCS 2019 to 2021 in terms of sociodemographic characteristics. For the items of the SUS and the total SUS score, means and SDs were calculated.

The medians, SDs, and IQRs of the food group, energy, and nutrient consumption assessed using both methods were calculated. Owing to skewed distributions, the nonparametric Wilcoxon signed rank test was used to test whether differences between the DitEetIk! app and the 24-hour dietary recalls for food group and nutrient intake were symmetrical around 0. Differences were considered relevant if the median intake was >10% of the 24-hour dietary recall value. Only food groups for which the 75th percentile was >0 were reported. The analyses were repeatedly stratified by educational level (3 classes) and BMI (3 classes).

In addition, the number of consumers and median intake for consumers only were calculated for each food group for each method. The McNemar test was used to test whether being a consumer per food group differed significantly between both methods. In addition, the Wilcoxon signed rank test was used to test whether the distribution of food group consumption only differed systematically by method. Spearman rank correlation coefficients were calculated for food group consumption and also energy and nutrient intake assessed using both methods.

Bland-Altman plots were constructed for the intake of energy and energy percentage derived from fat, protein, and carbohydrates, plotting the difference in intake assessed using both methods against the mean intake for each participant. The derived 95% limits of agreement [27] were presented to provide information on the variation in individual relative validity.

To assess any design effects, a nonparametric Wilcoxon signed rank test was used to test whether there was a systematic difference between the 24-hour dietary recalls in the relative validity study and the DNFCS 2019 to 2021 regarding the intake of energy, nutrients, and food groups.

All statistical analyses were conducted using SAS (version 9.4; SAS Institute Inc). *P* values of <.05 were considered statistically significant, and 2-sided statistical tests were performed.

## Results

### Study Population Characteristics

**Figure 2:**
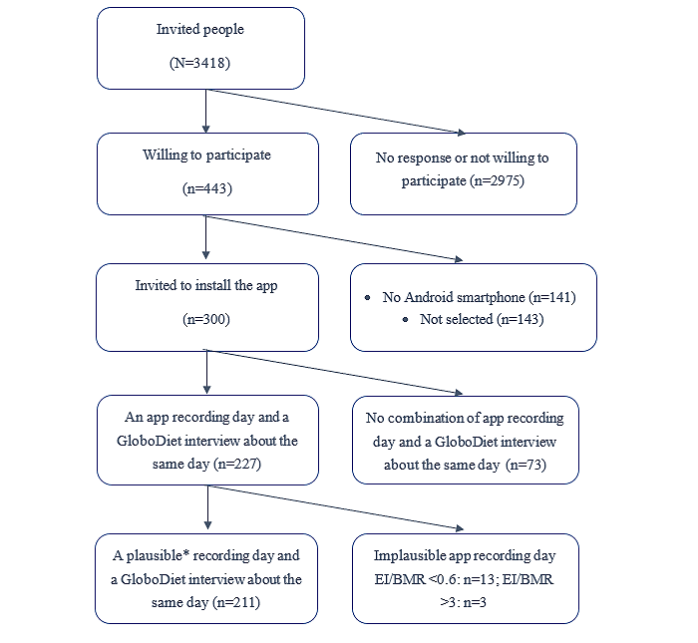
Participant recruitment and selection flow. A plausible recording day was a recording day with a ratio of energy intake over BMR between 0.6 and 3.0. BMR: basal metabolic rate; EI: energy intake.

Of the 3418 contacted people, 443 (12.96%) were willing to participate (Figure 2). A total of 300 people were invited to start the study based on their ability to use an Android smartphone and their sociodemographic characteristics. Of the 300 invited individuals, 227 (75.7%) completed a combination of a DitEetIk! app recording day and a 24-hour dietary recall interview for the same day. In total, 7% (16/227) of the participants were excluded because of an implausible ratio of energy intake to BMR of <0.6 or >3.0, resulting in 211 participants included in the analyses.

Approximately half (107/211, 50.7%) of the participants were women (Table 1). The study population consisted of more participants with a middle and higher educational level (81/211, 38.4% and 93/211, 44.1%, respectively) than those with a lower educational level (37/211, 17.5%). Of the 211 participants, 129 (61.1%) had a BMI of ≥25 kg/m^2^. Fewer people in the highest age category of 60 to 79 years participated in the study (58/211, 27.5%) than people in the 2 lower age categories (76/211, 36% and 77/211, 36.5%). The 211 participants included in the DitEetIk! app study were matched with 211 participants of the DNFCS 2019 to 2021. They had similar distributions in terms of gender, age, educational level, and BMI.

Of the 211 participants, 207 (98.1%) completed 3 DitEetIk! app recording days. Most GloboDiet 24-hour dietary recall interviews were for the first or second DitEetIk! app recording day (78/211, 37% and 84/211, 39.8%, respectively). Most of the participants started recording their food consumption on the DitEetIk! app in the morning (105/211, 49.8%) or afternoon (61/211, 28.9%) and ended their recording the same day in the evening (95/211, 45%) or the next day in the morning (82/211, 38.9%). The time between starting and ending the food recording using the DitEetIk! app was between 8 and 20 hours for 46% (97/211) of the participants, whereas it took >20 hours for 33.2% (70/211) of the participants and <8 hours for 20.9% (44/211) of the participants (Multimedia Appendix 1).

Food identification for recording was performed via text searching of the food items at the generic level for 23.6% (211/894) of the products and choosing previously selected food items (175/894, 19.6%). Branded food items were recorded via text searching (169/894, 18.9%) and barcode scanning (114/894, 12.8%). The option to make an individual recipe was used for 1.8% (16/894) of the products, associated food items (fats and oils for frying and milk or sugar in tea or coffee) were used for 16.4% (147/894) of the products, and supplements were used for 6.9% (62/894) of the products. Without considering previously selected food items, branded food items were recorded 39.4% (283/719) of times (Multimedia Appendix 2).

The SUS questionnaire was used to evaluate the system usability of the DitEetIk! app. The questionnaire was completed by 98.1% (207/211) of the included participants. The mean score per question ranged from 1.7 (SD 0.8) to 3.7 (SD 0.9). The mean overall score of the SUS was 66.6 (SD 15.1).

**Table 1:** Characteristics of the 211 participants in the DitEetIk! app study and matched participants from the Dutch National Food Consumption Survey (DNFCS) 2019 to 2021.

Characteristic		Evaluation study (n=211), n (%)	DNFCS 2019-2021 (n=211), n (%)
**Gender**			
	Men	104 (49.3)	104 (49.3)
	Women	107 (50.7)	107 (50.7)
**Age category (y)**			
	18-39	76 (36)	75 (35.5)
	40-59	77 (36.5)	78 (37)
	60-79	58 (27.5)	58 (27.5)
**Highest educational level attained**			
	Low^a^	37 (17.5)	31 (14.7)
	Middle^b^	81 (38.4)	87 (41.2)
	High^c^	93 (44.1)	93 (44.1)
**BMI category (kg/m^2^)**			
	<18.5	1 (0.5)	2 (0.9)
	≥18.5 to ≤25	81 (38.4)	81 (38.4)
	≥25 to ≤30	73 (34.6)	73 (34.6)
	≥30	56 (26.5)	55 (26.1)

^a^Low educational level: primary education, lower vocational education, or advanced elementary education.

^b^Middle educational level: intermediate vocational education or higher secondary education.

^c^High educational level: higher vocational education and university.

### Relative Validity for Food Groups

For 44% (7/16) of the food groups, no statistically significant differences between the median intake of food groups assessed using the DitEetIk! app food record and the 24-hour dietary recall were observed (Table 2). No statistically significant differences were observed for the median consumption of vegetables (*P*=.13), meat (*P*=.10), eggs (*P*=.44), nuts (*P*=.73), bread (*P*=.95), potatoes (*P*=.96), and snacks (*P*=.41).

Statistically significant differences of >10% between the 2 methods were observed for the median consumption of fruit (83 vs 130 g/d; *P*=.005), added fats (12 vs 17 g/d; *P*=.001), milk and milk products (219 vs 252 g/d; *P*=0.02), cereal products (6 vs 20 g/d; *P*=.01), and sauces (2 vs 22 g/d; *P*<.001). For all food groups except “Other,” the DitEetIk! app assessed a lower median consumption and, therefore, relatively underestimated the consumption of food groups compared with the 24-hour dietary recalls. However, when looking at the mean rather than the median, for 7 food groups, higher values were observed in the DitEetIk! app. Spearman correlation coefficients ranged from 0.50 for the food group “Other” to 0.88 for the food groups *Potatoes*, *Sandwich spreads*, and *Snacks*. The median correlation coefficient was 0.78.

A significant difference in the number of consumers between the 2 methods was observed for the food groups *Fruit* (*P*=.002), *Added fats* (*P*=.02), *Cereal products* (*P*=.03), *Sauces* (*P*<.001), and *Other products (P*<.001; Table 3). This list includes 4 of the 5 food groups for which differences in median intake for all participants were statistically significant and of >10%. Focusing on the 5 food groups, the median intake of consumers differed significantly for *Added fats* and *Sauces* (median 15 and 24 grams per day in the DitEetIk! app vs 20 and 45 grams per day in the GloboDiet 24-hour dietary recalls; *P*=.06 and *P*=.004) but not for *Fruit*, *Milk and milk products*, and *Cereal products*, whereas for the food groups *Fish* (90 vs 115 g/d), *Cheese* (36 vs 45 g/d), *Sandwich spreads* (26 vs 20 g/d), and *Soups* (188 vs 50 g/d), significant differences of >10% were also observed in the median intake of consumers only.

Within the strata of educational level (3 classes), a few significant differences that were ≥10% for the median were observed for food groups (Multimedia Appendix 1). For sauces (low educational level *P*=.02; middle educational level *P*=.006; high educational level *P*<.001), the differences were consistent across the 3 educational levels. For milk and milk products, only those with a high educational level had a significantly lower median in the DitEetIk! app compared with the GloboDiet 24-hour dietary recalls (*P*=.01). Within the strata of BMI (3 levels), more differences were observed, but there was no consistent pattern in which differences were generally smaller or larger for persons in one of the BMI classes (Multimedia Appendix 2).

**Table 2:** The mean, SD, median, and IQR of consumption of food groups^a^ as assessed using the DitEetIk! app and 24-hour dietary recalls for the same day and their correlation for the 211 participants with plausible energy intakes.

Food group	DitEetIk! app food record (g/d)		GloboDiet 24-hour dietary recall (g/d)		Wilcoxon signed rank test *P* value^b^	Spearman correlation coefficient
	Values, mean (SD)	Values, median (IQR)	Values, mean (SD)	Values, median (IQR)		
Vegetables	163 (200)	117 (31-226)	160 (144)	130 (50-240)	.13	0.76
Fruit	128 (186)	83 (0-188)	140 (146)	130 (0-217)	.005	0.79
Added fats	16 (16)	12 (3-24)	19 (15)	17 (6-29)	.001	0.54
Meat	103 (112)	73 (23-135)	92 (83)	75 (33-120)	.10	0.70
Eggs	17 (37)	0 (0-0)	17 (34)	0 (0-13)	.44	0.76
Nuts	15 (30)	0 (0-20)	15 (30)	0 (0-20)	.73	0.84
Milk (products)	264 (263)	219 (16-391)	288 (248)	252 (80-423)	.02	0.80
Cheese	33 (36)	30 (0-56)	39 (44)	31 (0-62)	.006	0.76
Bread	146 (113)	126 (70-199)	138 (88)	132 (70-180)	.95	0.85
Cereal products	67 (133)	6 (0-88)	74 (106)	20 (0-119)	.01	0.80
Potatoes	72 (119)	0 (0-128)	66 (104)	0 (0-120)	.96	0.88
Drinks	1888 (956)	1836 (1275-2311)	2097 (889)	1963 (1582-2539)	<.001	0.68
Sandwich spreads	15 (27)	0 (0-20)	12 (23)	0 (0-15)	.05	0.88
Snacks	91 (119)	52 (15-118)	83 (89)	56 (14-126)	.41	0.88
Sauces	21 (37)	2 (0-26)	33 (38)	22 (0-57)	<.001	0.60
Other	13 (52)	0 (0-10)	5 (12)	0 (0-5)	<.001	0.50

^a^Food groups are Wheel of Five food groups—main groups [23]. The food groups *Fish*, *Legumes*, and *Soups* were excluded as the 75th percentile was 0 for both methods. Table 3 provides more information on these food groups.

^b^Wilcoxon signed rank test (normal approximation) of the differences between intake assessed using the DitEetIk! app and the GloboDiet 24-hour dietary recalls for the same day.

**Table 3:** Number of consumers of a food group and median of consumed amount of a food group^a^ for consumers only as assessed using the DitEetIk! app and the 24-hour dietary recalls (n=211).

Food group	DitEetIk! app food record (g/d)			GloboDiet 24-hour dietary recall (g/d)			Mc Nemar test *P* value^b^		Wilcoxon signed rank test *P* value^c^
	Consumers (n=211), n (%)	Consumers, median	Consumers (n=211), n (%)		Consumers, median				
Vegetables	175 (82.9)	146	181 (85.8)		147	.16		.23	
Fruit	134 (63.5)	156	148 (70.1)		165	.002		.21	
Added fats	173 (82)	15	186 (88.2)		20	.02		.06	
Fish	25 (11.8)	90	26 (12.3)		115	.66		.04	
Legumes	5 (2.4)	111	10 (4.7)		79	.06		.38	
Meat	178 (84.4)	86	179 (84.8)		85	.81		.06	
Eggs	52 (24.6)	50	61 (28.9)		50	.08		.83	
Nuts	66 (31.3)	32	70 (33.2)		31	.32		.29	
Milk and milk products	165 (78.2)	265	173 (82)		302	.06		.13	
Cheese	138 (65.4)	36	145 (68.7)		45	.11		.03	
Bread	194 (91.9)	136	198 (93.8)		140	.10		.71	
Cereal products	113 (53.6)	70	124 (58.8)		98	.03		.05	
Potatoes	87 (41.2)	150	90 (42.7)		142	.44		.91	
Drinks	206 (97.6)	1848	211 (100)		1963	N/A^d^		<.001	
Sandwich spreads	85 (40.3)	26	89 (42.2)		20	.25		.01	
Soups	33 (15.6)	188	28 (13.3)		50	.10		<.001	
Snacks	173 (82)	72	172 (81.5)		78	.76		.69	
Sauces	116 (55)	24	143 (67.8)		45	<.001		.004	
Other	126 (59.7)	5	59 (28)		12	<.001		.11	

^a^Food groups are Wheel of Five food groups [23].

^b^McNemar test of the differences between the number of consumers of the DitEetIk! app and the GloboDiet 24-hour dietary recalls for the same day.

^c^Wilcoxon signed rank test (normal approximation) of the differences between intake assessed using the DitEetIk! app and the GloboDiet 24-hour dietary recalls for the same day for consumers of each food group.

^d^N/A: not applicable.

### Relative Validity for Nutrients

For 41% (12/29) of the nutrients, the median intake assessed using the DitEetIk! app and the 24-hour dietary recalls did not differ significantly (Table 4). Statistically significant differences of >10% between the 2 methods were observed for the median intake of vitamin A (453 vs 515 µg retinol activity equivalents/d; *P*<.001), folate (equivalents; 241 vs 269 µg/d; *P*<.001), vitamin D (1.8 vs 2.1 µg/d; *P*<.001), and vitamin E (9.7 vs 11.7 mg/d; *P*<.001). For 8 nutrients, significant differences were between 5% and 10%, and for 5 nutrients, they were <5%. With the exceptions of mono- and disaccharides and magnesium, in all cases of statistically significant differences, the DitEetIk! app had lower values than the 24-hour dietary recalls. Spearman correlation coefficients between intake assessed using the DitEetIk! app and the 24-hour dietary recalls ranged from 0.55 for sodium to 0.9 for alcohol, with a median correlation coefficient of 0.72.

Compared with the expected energy intake, the mean underreporting using the DitEetIk! app was 19.9% (0.316/1.59; calculated as [measured-expected physical activity level]/[expected physical activity level]) versus 20.1% (0.321/1.59) using the 24-hour dietary recalls. At the individual level, 20.4% (43/211) of the participants could be considered to be underreporting and 0.5% (1/211) of the participants could be considered to be overreporting energy intake using the DitEetIk! app. For the 24-hour dietary recalls, 18% (38/211) of the participants could be considered to be underreporting, and none were overreporting.

**Figure 3:**
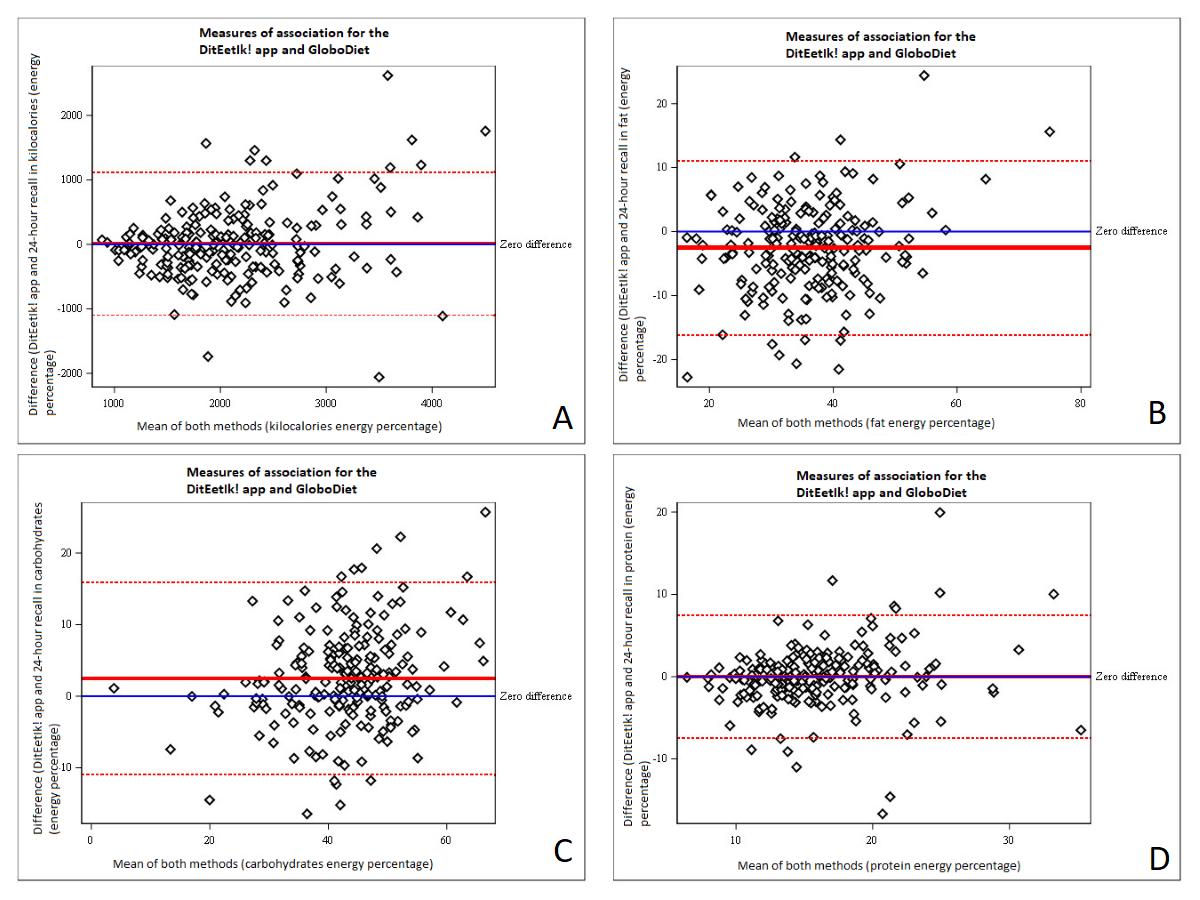
Bland-Altman plot assessed using the DitEetIk! app and GloboDiet (GD) 24-hour dietary recalls (n=211). (A) Results for energy intake, (B) results for fat (energy percentage), (C) results for carbohydrates (energy percentage), and (D) results for protein (energy percentage).

Figure 3 shows the Bland-Altman plots for energy intake and for fat, carbohydrates, and protein expressed as a percentage of energy intake. The mean differences and 95% limits of agreement were 14 (−1096 to 1124) for energy in kilocalories, −2.6 (−16.2 to 11) for energy percentage derived from fat, 2.5 (−10.9 to 16) for energy percentage derived from carbohydrates, and −7.5 (−7.5 to 7.4) for energy percentage derived from protein.

**Table 4:** The mean, SD, median, and IQR of energy and nutrient intake per day as assessed using the DitEetIk! app and the 24-hour dietary recalls and their correlation (n=211).

Nutrients	DitEetIk! app food record			GloboDiet 24-hour dietary recall			Wilcoxon signed rank test *P* value^a^		Spearman correlation coefficient
	Values, mean (SD)	Values, median (IQR)	Values, mean (SD)		Values, median (IQR)				
Energy (kcal)	2112 (795)	1994 (1534-2488)	2100 (675)		1994 (1614-2539)	.59		0.75	
Fat (g)	83 (42)	76 (51-105)	88 (39)		81 (61-110)	.001		0.74	
Saturated fatty acids (g)	29 (15)	27 (18-38)	31 (15)		28 (20-39)	.01		0.70	
Protein (g)	82 (34)	77 (59-100)	82 (30)		77 (62-101)	.54		0.70	
Vegetable protein (g)	33 (15)	30 (23-41)	33 (13)		30 (23-42)	.37		0.75	
Carbohydrates (g)	230 (99)	215 (166-280)	215 (77)		208 (160-259)	.04		0.78	
Mono- and disaccharides (g)	93 (53)	81 (56-120)	85 (43)		80 (56-111)	.01		0.80	
Fiber (g)	21 (11)	20 (14-26)	21 (9)		20 (14-26)	.15		0.75	
Alcohol (g)	10 (21)	0 (0-11)	9 (20)		0 (0-10)	.45		0.92	
Water (g)	2681 (1012)	2570 (2048-3229)	2907 (955)		2775 (2285-3349)	<.001		0.68	
Vitamin A (µg RAE^b^)	714 (1007)	453 (286-685)	804 (1013)		515 (334-806)	<.001		0.74	
Vitamin B1 (mg)	1.0 (0.6)	0.9 (0.7-1.3)	1.0 (0.5)		0.9 (0.7-1.3)	.21		0.63	
Vitamin B2 (mg)	1.3 (0.6)	1.3 (0.9-1.6)	1.4 (0.6)		1.3 (1.0-1.8)	.002		0.73	
Vitamin B3 (mg)	18.8 (10.8)	16.5 (10.8-24.4)	19.0 (9.3)		17.3 (11.5-24.4)	.17		0.70	
Vitamin B6 (mg)	1.5 (0.7)	1.4 (1.0-1.9)	1.6 (0.7)		1.5 (1.1-1.9)	<.001		0.71	
Folate (equivalents; µg)	266 (148)	241 (172-331)	286 (122)		269 (203-357)	<.001		0.67	
Vitamin B12 (µg)	3.8 (3.2)	2.9 (2.0-4.8)	4.2 (3.4)		3.2 (2.2-5.1)	.001		0.78	
Vitamin C (mg)	94 (123)	65 (32-108)	88 (74)		67 (33-128)	.09		0.77	
Vitamin D (µg)	2.3 (2.2)	1.8 (0.9-2.9)	2.7 (2.5)		2.1 (1.2-3.5)	<.001		0.64	
Vitamin E (mg)	11.4 (7.3)	9.7 (6.5-14.3)	13.1 (6.7)		11.7 (8.2-16.7)	<.001		0.58	
Calcium (mg)	965 (451)	909 (658-1231)	1006 (452)		921 (664-1295)	.12		0.74	
Iodine (µg)^c^	173 (92)	159 (111-213)	172 (79)		162 (116-209)	.42		0.80	
Iron (mg)	10.3 (4.5)	9.3 (7.2-12.7)	10.2 (4.0)		9.4 (7.2-12.1)	.41		0.73	
Magnesium (mg)	338 (126)	334 (249-403)	346 (118)		328 (264-411)	.04		0.72	
Phosphorus (mg)	1504 (537)	1466 (1086-1864)	1506 (489)		1478 (1135-1790)	.63		0.72	
Potassium (mg)	3202 (1202)	3147 (2314-3860)	3206 (1059)		3106 (2475-3886)	.38		0.72	
Selenium (µg)	52 (44)	43 (31-58)	50 (32)		43 (31-63)	.60		0.68	
Sodium (mg)^c^	2625 (1443)	2373 (1637-3096)	2410 (1040)		2283 (1694-2875)	.05		0.55	
Zinc (mg)	10.1 (4.5)	9.4 (7.0-12.5)	10.3 (3.9)		10.0 (7.5-12.3)	.10		0.70	

^a^Wilcoxon signed rank test (normal approximation) of the differences between intake assessed using the DitEetIk! app and the GloboDiet 24-hour dietary recalls for the same day.

^b^RAE: retinol activity equivalents.

^c^Sodium and iodine from food only.

### Evaluation of the GloboDiet 24-Hour Dietary Recall Data

For almost all food groups, the consumption of participants in the DitEetIk! app evaluation study, assessed using the GloboDiet 24-hour dietary recall, and the consumption of matched participants selected from the DNFCS 2019 to 2021 did not differ significantly (Table 5). Statistically significant differences of >10% in median intake between the 2 methods were only observed for the consumption of milk and milk products (median 252 grams per day in the DitEetIk! app vs 282 grams per day in the GloboDiet 24-hour dietary recalls; *P*=.03) and bread (median 132 grams per day vs 105 grams per day; *P*=.03).

For all nutrients except vitamin B3, the intake of participants in the evaluation study, assessed using the GloboDiet 24-hour dietary recalls, and the intake of the matched participants selected from the DNFCS 2019 to 2021 did not differ significantly (Table 6).

**Table 5:** Comparison of consumption of food groups assessed using the GloboDiet 24-hour dietary recalls in the DitEetIk! app evaluation study and the first interview in the Dutch National Food Consumption Survey (DNFCS) 2019 to 2021 for a matched group of participants (n=211).

Food group^a^	DitEetIk! app evaluation study (g/d)			DNFCS 2019-2021 (g/d)			*P* value^b^
	Values, mean (SD)	Values, median (IQR)	Values, mean (SD)		Values, median (IQR)		
Vegetables	160 (144)	130 (50-240)	155 (140)		125 (53-217)	>.99	
Fruit	140 (146)	130 (0-217)	124 (135)		108 (0-195)	.51	
Added fats	19 (15)	17 (6-29)	22 (20)		18 (8-32)	.006	
Fish	17 (57)	0 (0-0)	15 (44)		0 (0-0)	.91	
Legumes	4 (20)	0 (0-0)	8 (36)		0 (0-0)	.09	
Meat	92 (83)	75 (33-120)	88 (80)		77 (29-116)	.76	
Eggs	17 (34)	0 (0-13)	16 (32)		0 (0-13)	.85	
Nuts	15 (30)	0 (0-20)	19 (45)		0 (0-22)	.24	
Milk and milk products	288 (248)	252 (80-423)	332 (267)		282 (150-484)	.03	
Cheese	39 (44)	31 (0-62)	38 (39)		30 (0-62)	.85	
Bread	138 (88)	132 (70-180)	117 (80)		105 (60-169)	.03	
Cereal products	74 (106)	20 (0-119)	79 (108)		30 (0-122)	.57	
Potatoes	66 (104)	0 (0-120)	69 (93)		0 (0-140)	.26	
Drinks	2097 (889)	1963 (1582-2539)	2132 (914)		1958 (1468-2608)	.63	
Sandwich spreads	12 (23)	0 (0-15)	18 (29)		0 (0-23)	.03	
Soups	12 (46)	0 (0-0)	17 (66)		0 (0-0)	.57	
Snacks	83 (89)	56 (14-126)	71 (79)		41 (10-114)	.24	
Sauces	33 (38)	22 (0-57)	29 (44)		11 (0-36)	.14	
Other	5 (12)	0 (0-5)	6 (16)		0 (0-5)	.75	

^a^Food groups are Wheel of Five food groups [23].

^b^Wilcoxon signed rank test (normal approximation) of the differences between intake assessed using GloboDiet 24-hour dietary recalls in the DitEetIk! app evaluation study and the first interview with adults in the DNFCS 2019 to 2021.

**Table 6:** Comparison of intake of energy and selected nutrients assessed using the GloboDiet 24-hour dietary recalls in the DitEetIk! app evaluation study and the first interview with adults in the Dutch National Food Consumption Survey (DNFCS) 2019 to 2021 (n=211).

Nutrients	DitEetIk! app food record		GloboDiet 24-hour dietary recall			*P* value^a^
	Values, mean (SD)	Values, median (IQR)	Values, mean (SD)	Values, median (IQR)		
Energy (kcal)	2100 (675)	1994 (1614-2539)	2091 (761)	2036 (1506-2535)	.86	
Fat (g)	88 (39)	81 (61-110)	89 (43)	81 (60-111)	.86	
Saturated fatty acids (g)	31 (15)	28 (20-39)	32 (16)	30 (22-40)	.48	
Protein (g)	82 (30)	77 (62-101)	80 (29)	77 (62-100)	.29	
Vegetable protein (g)	33 (13)	30 (23-42)	32 (14)	30 (22-39)	.31	
Carbohydrates (g)	215 (77)	208 (160-259)	217 (88)	203 (155-262)	.71	
Mono- and disaccharides (g)	85 (43)	80 (56-111)	94 (55)	85 (60-112)	.21	
Fiber (g)	21 (9)	20 (14-26)	22 (9)	20 (16-26)	.18	
Alcohol (g)	9 (20)	0 (0-10)	8 (15)	0 (0-12)	.64	
Water (g)	2907 (955)	2775 (2285-3349)	2950 (962)	2831 (2235-3481)	.75	
Vitamin A (µg RAE^b^)	804 (1013)	515 (334-806)	828 (1026)	570 (360-894)	.42	
Vitamin B1 (mg)	1.0 (0.5)	0.9 (0.7-1.3)	1.0 (0.6)	0.9 (0.6-1.2)	.96	
Vitamin B2 (mg)	1.4 (0.6)	1.3 (1.0-1.8)	1.4 (0.7)	1.4 (1.0-1.8)	.79	
Vitamin B3 (mg)	19.0 (9.3)	17.3 (11.5-24.4)	17.1 (9.3)	15.7 (10.6-21.2)	.02	
Vitamin B6 (mg)	1.6 (0.7)	1.5 (1.1-1.9)	1.5 (0.7)	1.4 (1.1-1.9)	.29	
Folate (equivalents; µg)	286 (122)	269 (203-357)	272 (121)	243 (194-340)	.13	
Vitamin B12 (µg)	4.2 (3.4)	3.2 (2.2-5.1)	4.3 (3.1)	3.6 (2.6-5.6)	.65	
Vitamin C (mg)	88 (74)	67 (33-128)	82 (65)	65 (35-110)	.39	
Vitamin D (µg)	2.7 (2.5)	2.1 (1.2-3.5)	2.7 (2.1)	2.2 (1.1-3.9)	.52	
Vitamin E (mg)	13.1 (6.7)	11.7 (8.2-16.7)	13.5 (8.4)	12.1 (8.4-16.8)	.85	
Calcium (mg)	1006 (452)	921 (664-1295)	1045 (480)	983 (702-1321)	.39	
Iodine (µg)^c^	172 (79)	162 (116-209)	166 (75)	158 (112-210)	.42	
Iron (mg)	10.2 (4.0)	9.4 (7.2-12.1)	10.6 (4.1)	10.0 (7.9-12.5)	.35	
Magnesium (mg)	346 (118)	328 (264-411)	349 (128)	325 (250-426)	.86	
Phosphorus (mg)	1506 (489)	1478 (1135-1790)	1493 (518)	1490 (1128-1781)	.67	
Potassium (mg)	3206 (1059)	3106 (2475-3886)	3223 (1127)	3170 (2358-3768)	.99	
Selenium (µg)	50 (32)	43 (31-63)	51 (46)	41 (30-57)	.40	
Sodium (mg)^c^	2410 (1040)	2283 (1694-2875)	2321 (1042)	2125 (1562-2983)	.42	
Zinc (mg)	10.3 (3.9)	10.0 (7.5-12.3)	10.5 (4.8)	10.2 (7.6-13.1)	.53	

^a^Wilcoxon signed rank test (normal approximation) of the differences between intake assessed using the GloboDiet 24-hour dietary recalls in the DitEetIk! app evaluation study and the first interview with adults in the DNFCS 2019 to 2021.

^b^RAE: retinol activity equivalents.

^c^Sodium and iodine from food only.

## Discussion

### Principal Findings

Compared with GloboDiet 24-hour dietary recalls, the DitEetIk! app assessed similar mean levels of energy intake but somewhat lower median levels of intake for several food groups and nutrients. Differences were of >10% for fruit; added fats; cereal products; sauces; and vitamins A, D, and E and folate. Of all logged food items and beverages, most were selected via text searching, whereas the scanning functionality was used for approximately one-seventh of the food products.

Incomplete recording of consumed food items in the DitEetIk! app seems to have occurred for various food groups, such as fruit, added fats, cereal products, and sauces. During the GloboDiet 24-hour dietary recall, the trained interviewer specifically probes for easily forgotten food items [28], which may explain this difference. Similarly, other studies evaluating mobile food record apps based on text searching reported food omissions, particularly of condiment food items [29]. In a study using wearable camera images as a reference, it was observed that the most forgotten food groups in the Australian Eat and Track app were savory sauces and condiments, vegetables, confectionery, fruit and dairy, and alternatives [30]. There are various options to stimulate complete recording. According to a review of smartphone dietary assessment tools, the most common feature to do so was to allow participants to review the records and make adjustments if information was missing or false [31]. This feature was also built into the DitEetIk! app; before submitting the food recording for one day, participants were shown an overview of reported food items and were asked whether this was complete. In addition, incorporating (more) probing questions for frequently omitted food items into DitEetIk! app could be a way to remind a participant to report such food items. This could be probing questions either linked to other food items (eg, salad dressing in the case of salads) or linked to eating occasions (eg, fruit in between meals). Alternatively, sending prompts when an eating moment is expected or when the DitEetIk! app has not been used for a certain period or allowing participants to explicitly state that they did not consume anything at a given eating occasion are also approaches with the potential to improve completeness [32].

In contrast to the results for median intake, for some food groups and nutrients, the mean intake was higher in the DitEetIk! app than in the 24-hour dietary recalls. For the food groups *Vegetables*, *Meat*, *Bread*, and *Potatoes*, this was caused by higher amounts recorded in the DitEetIk! app than those indicated in the 24-hour dietary recalls. Choosing unlikely high portion sizes in the DitEetIk! app was possible without a warning message in case the amount eaten was indicated in units rather than grams, whereas in the GloboDiet 24-hour dietary recalls, all indicated portions were converted into grams and checked against set improbable maximum values, and if needed, the interviewer was prompted to check with the participant whether the answer was correct [28]. A similar functionality could be considered for inclusion in the DitEetIk! app.

Of all logged food and beverages, most were selected via text searching. In the feedback given by participants in the remark field of the DitEetIk! app, they mentioned that finding the correct food item on the list was a challenging task. This disadvantage of extensive food item lists was also described in a systematic review [33]. This was probably the reason why the average SUS score for the DitEetIk! app was just below 70, the threshold that is generally considered “good” [34]. Only 12.8% (114/894) of all logged food items and drinks were scanned. This percentage was lower than expected based on experiences in a project in which approximately 50% of the food items were scanned using a commercial smartphone food record (personal communication by MO). The availability of branded food items in the DitEetIk! app was still limited, and these did not include food items from some supermarket chains or that were not matched to the generic food composition database. This may have affected the use of the scanning option. One could understand that participants stopped scanning barcodes after some failed attempts. Therefore, including more branded food items in the DitEetIk! app is highly recommended. In addition, a crowdsourcing function could be incorporated whereby users can contribute information on missing products, such as that developed for the FoodSwitch app [35]. The collected food product information can then be added to the database to ensure that the DitEetIk! app is supported by actual and complete product information. If more food items are scanned, food recording will probably be perceived as easier.

As described previously, based on the main findings, several options for improvement via additional DitEetIk! app functionalities can be formulated. However, one should also be careful not to burden participants with too frequent notifications, reminders, and prompts [33]. More insight on the impact of different features used in smartphone-based dietary assessment tools and the characteristics of these features on the respondents’ willingness and ability to record intake reliably and on the validity of the recorded dietary data is needed [31].

### Strengths and Limitations

This study is one of the few food record validation studies (Zhang et al [29]) that report results for a rather comprehensive list of food groups and nutrients in a group of >200 men and women of various ages. An important limitation is that relative rather than objective validity was studied. Therefore, lower and higher values compared with GloboDiet 24-hour dietary recall values cannot be interpreted as underestimation or overestimation. However, the results on energy misreporting were included, which are not dependent on the subjective reporting of dietary intake. In the future, follow-up validation with doubly labeled water and excretion of nitrogen, potassium, and sodium in 24-hour urine is recommended. Another limitation is the large number of statistical tests that were conducted, which may have led to chance findings. Moreover, the study did not follow a crossover design, which might have caused a potential memory or learning effect in the 24-hour dietary recall data. However, comparing these data with those from the DNFCS 2019 to 2021 gave no indication that this occurred. People with a lower education and of higher age were included in the study population but were underrepresented. We cannot conclude whether the relative validity is similar for these population subgroups. This study did not focus on the potential selection bias of including only participants who had an Android smartphone. Such an evaluation is also important for use in a national food consumption survey. Potentially, developing an iOS version of the DitEetIk! app and offering an interview option to persons without a smartphone needs to be considered. In this study, the DitEetIk! app was described according to the recommendations of Eldridge et al [11], and the validity study was reported according to the guidance provided by Kirkpatrick et al [26].

### Comparison With Prior Work

Although many different smartphone-based dietary assessment tools exist, only a few validation studies have been conducted. Burrows et al [36] concluded that their validity seems to be comparable with that of more traditional dietary assessment methods and that energy intake is often underreported. In a review from 2013 to 2019, Zhang et al [29] identified 14 smartphone-based food records that were not image based. In the meta-analyses based on 11 tools, all of them underestimated energy intake, with a pooled effect of approximately −200 kcal and limits of agreement of 1918 kcal. The results for the DitEetIk! app fit with this picture, with a below-average mean underestimation and similar limits of agreement for energy intake. The relative validity results were also in line with those of Eetmeter, another Dutch app, although the food groups for which underestimation occurred partly differed [20]. Unlike the DitEetIk! app, Eetmeter shows energy and nutrient values for logged food items, which might influence reporting.

### Conclusions

Compared with GloboDiet 24-hour dietary recalls, the DitEetIk! app assessed somewhat lower levels of intake for several food groups and nutrients. Therefore, adding functionalities to the DitEetIk! app that stimulate more complete food recording is important before using the app in national food consumption surveys. In addition, it is advisable to develop a functionality to warn participants when entering extremely large consumption amounts. Less participant burden and more detailed information about consumed food items can be obtained by stimulating the use of barcode scanning.

## Appendix

Multimedia Appendix 1 The mean, SD, median, and IQR of consumption of food groups (g/d) by educational level as assessed using the DitEetIk! app and 24-hour dietary recalls for the same day and their correlation for 211 participants with plausible energy intakes.

Multimedia Appendix 2 The mean, SD, median, and IQR of consumption of food groups (g/d) by BMI as assessed using the DitEetIk! app and 24-hour dietary recalls for the same day and their correlation for 211 participants with plausible energy intakes.

## Abbreviations

BMR: basal metabolic rate
DNFCS: Dutch National Food Consumption Survey
LEDA: Dutch-branded food database (Levensmiddelendatabank)
SUS: System Usability Scale

## Data Availability

The data presented in this paper are available upon request from the corresponding author. The data are not publicly available to comply with the requirements of the General Data Protection Regulation (European Union) 2016/679.
