# Table Correction: A Smartphone Food Record App Developed for the Dutch National Food Consumption Survey: Relative Validity Study

**DOI:** 10.2196/59530

**Published:** 2024-04-26

**Authors:** Marga Ocké, Ceciel Simone Dinnissen, Coline van den Bogaard, Marja Beukers, José Drijvers, Eline Sanderman-Nawijn, Caroline van Rossum, Ido Toxopeus

**Affiliations:** 1 National Institute for Public Health and the Environment Bilthoven Netherlands

In “A Smartphone Food Record App Developed for the Dutch National Food Consumption Survey: Relative Validity Study” (JMIR Mhealth Uhealth 2024;12:e50196) the authors noted the following errors in Tables 2 and 5:

Due to an unintended line alignment issue, all values in the *DitEetIk! app food record* and *GloboDiet 24-hour dietary recall (g/d)* columns of Table 2 were incorrect for the following rows: *Meat, Eggs, Nuts, Milk (products), Cheese, Bread, Cereal products, Potatoes, Drinks, Sandwich spreads, Snacks, Sauces*, and *Other.*

Table 2 has been corrected as follows:

**Table 2:** The mean, SD, median, and IQR of consumption of food groups^a^ as assessed using the DitEetIk! app and 24-hour dietary recalls for the same day and their correlation for the 211 participants with plausible energy intakes.

Food group	DitEetIk! app food record (g/d)		GloboDiet 24-hour dietary recall (g/d)		Wilcoxon signed rank test *P* value^b^	Spearman correlation coefficient
	Values, mean (SD)	Values, median (IQR)	Values, mean (SD)	Values, median (IQR)		

Vegetables	163 (200)	117 (31-226)	160 (144)	130 (50-240)	.13	0.76
Fruit	128 (186)	83 (0-188)	140 (146)	130 (0-217)	.005	0.79
Added fats	16 (16)	12 (3-24)	19 (15)	17 (6-29)	.001	0.54
Meat	103 (112)	73 (23-135)	92 (83)	75 (33-120)	.10	0.70
Eggs	17 (37)	0 (0-0)	17 (34)	0 (0-13)	.44	0.76
Nuts	15 (30)	0 (0-20)	15 (30)	0 (0-20)	.73	0.84
Milk (products)	264 (263)	219 (16-391)	288 (248)	252 (80-423)	.02	0.80
Cheese	33 (36)	30 (0-56)	39 (44)	31 (0-62)	.006	0.76
Bread	146 (113)	126 (70-199)	138 (88)	132 (70-180)	.95	0.85
Cereal products	67 (133)	6 (0-88)	74 (106)	20 (0-119)	.01	0.80
Potatoes	72 (119)	0 (0-128)	66 (104)	0 (0-120)	.96	0.88
Drinks	1888 (956)	1836 (1275-2311)	2097 (889)	1963 (1582-2539)	<.001	0.68
Sandwich spreads	15 (27)	0 (0-20)	12 (23)	0 (0-15)	.05	0.88
Snacks	91 (119)	52 (15-118)	83 (89)	56 (14-126)	.41	0.88
Sauces	21 (37)	2 (0-26)	33 (38)	22 (0-57)	<.001	0.60
Other	13 (52)	0 (0-10)	5 (12)	0 (0-5)	<.001	0.50

^a^Food groups are Wheel of Five food groups—main groups [23]. The food groups *Fish*, *Legumes*, and *Soups* were excluded as the 75th percentile was 0 for both methods. Table 3 provides more information on these food groups.

^b^Wilcoxon signed rank test (normal approximation) of the differences between intake assessed using the DitEetIk! app and the GloboDiet 24-hour dietary recalls for the same day.

Similarly, due to an unintended line alignment issue, all values in the *DitEetIk! app evaluation study (g/d)* and *DNFCS 2019-2021 (g/d)* columns of Table 5 were incorrect for the following rows: *Meat, Eggs, Nuts, Milk and milk products, Cheese, Bread, Cereal products, Potatoes, Drinks, Sandwich spreads, Soups, Snacks, Sauces*, and *Other*.

Table 5 has been corrected as follows:

**Table 5:** Comparison of consumption of food groups assessed using the GloboDiet 24-hour dietary recalls in the DitEetIk! app evaluation study and the first interview in the Dutch National Food Consumption Survey (DNFCS) 2019 to 2021 for a matched group of participants (n=211).

Food group^a^	DitEetIk! app evaluation study (g/d)		DNFCS 2019-2021 (g/d)		*P* value^b^
	Values, mean (SD)	Values, median (IQR)	Values, mean (SD)	Values, median (IQR)	

Vegetables	160 (144)	130 (50-240)	155 (140)	125 (53-217)	>.99
Fruit	140 (146)	130 (0-217)	124 (135)	108 (0-195)	.51
Added fats	19 (15)	17 (6-29)	22 (20)	18 (8-32)	.006
Fish	17 (57)	0 (0-0)	15 (44)	0 (0-0)	.91
Legumes	4 (20)	0 (0-0)	8 (36)	0 (0-0)	.09
Meat	92 (83)	75 (33-120)	88 (80)	77 (29-116)	.76
Eggs	17 (34)	0 (0-13)	16 (32)	0 (0-13)	.85
Nuts	15 (30)	0 (0-20)	19 (45)	0 (0-22)	.24
Milk and milk products	288 (248)	252 (80-423)	332 (267)	282 (150-484)	.03
Cheese	39 (44)	31 (0-62)	38 (39)	30 (0-62)	.85
Bread	138 (88)	132 (70-180)	117 (80)	105 (60-169)	.03
Cereal products	74 (106)	20 (0-119)	79 (108)	30 (0-122)	.57
Potatoes	66 (104)	0 (0-120)	69 (93)	0 (0-140)	.26
Drinks	2097 (889)	1963 (1582-2539)	2132 (914)	1958 (1468-2608)	.63
Sandwich spreads	12 (23)	0 (0-15)	18 (29)	0 (0-23)	.03
Soups	12 (46)	0 (0-0)	17 (66)	0 (0-0)	.57
Snacks	83 (89)	56 (14-126)	71 (79)	41 (10-114)	.24
Sauces	33 (38)	22 (0-57)	29 (44)	11 (0-36)	.14
Other	5 (12)	0 (0-5)	6 (16)	0 (0-5)	.75

^a^Food groups are Wheel of Five food groups [23].

^b^Wilcoxon signed rank test (normal approximation) of the differences between intake assessed using GloboDiet 24-hour dietary recalls in the DitEetIk! app evaluation study and the first interview with adults in the DNFCS 2019 to 2021.

The correction will appear in the online version of the paper on the JMIR Publications website on April 26, 2024, together with the publication of this correction notice. Because this was made after submission to PubMed, PubMed Central, and other full-text repositories, the corrected article has also been resubmitted to those repositories.

