# Supplementary material for: A Smartphone Food Record App Developed for the Dutch National Food Consumption Survey: Relative Validity Study
Source: JMIR Mhealth Uhealth. 2024 Feb 9;12:e50196. doi: 10.2196/50196 (PMC10891498; doi:10.2196/50196)
Supplement: Multimedia Appendix 1 [file mhealth_v12i1e50196_app1.docx]

Multimedia Appendix 1. The mean, median and IQR of consumption of food groups (grams per day) by educational level as assessed using the DitEetIk! app and 24-hour dietary recalls for the same day and their correlation for 211 participants with plausible energy intakes.

| Food group^a^ | Educational level^b^ | DitEetIk! app food record (grams per day) | | GloboDiet 24-hour dietary recall (grams per day) | | Wilcoxon signed rank test P value^c^ | Spearman correlation coefficient |
| --- | --- | --- | --- | --- | --- | --- | --- |
|  |  | Mean (SD) | Median (IQR) | Mean (SD) | Median (IQR) |  |  |
|  |  |  |  |  |  |  |  |
| Vegetables | Low | 182 (181) | 146 (71-244) | 156 (129) | 140 (48-238) | .97 | 0.76 |
| Vegetables | Middle | 136 (226) | 93 (24-189) | 132 (113) | 110 (51-180) | .11 | 0.73 |
| Vegetables | High | 178 (183) | 138 (28-253) | 187 (167) | 146 (52-282) | .40 | 0.77 |
| Fruit | Low | 110 (106) | 95 (0-178) | 138 (114) | 135 (1-198) | .18 | 0.66 |
| Fruit | Middle | 143 (251) | 54 (0-209) | 128 (125) | 126 (0-227) | .21 | 0.84 |
| Fruit | High | 122 (139) | 90 (0-188) | 150 (173) | 130 (0-200) | .06 | 0.80 |
| Added fats | Low | 15 (12) | 15 (1-24) | 22 (17) | 20 (9-37) | <.001 | 0.67 |
| Added fats | Middle | 13 (13) | 9 (2-18) | 17 (13) | 13 (5-26) | .04 | 0.35 |
| Added fats | High | 19 (19) | 13 (5-25) | 20 (16) | 18 (7-29) | .26 | 0.64 |
| Meat | Low | 136 (146) | 81 (48-176) | 108 (107) | 79 (33-156) | .03 | 0.85 |
| Meat | Middle | 90 (118) | 54 (20-106) | 80 (76) | 63 (26-113) | .56 | 0.74 |
| Meat | High | 101 (88) | 76 (39-131) | 96 (78) | 79 (45-124) | .58 | 0.59 |
| Eggs | Low | 17 (40) | 0 (0-0) | 17 (39) | 0 (0-0) | .69 | 0.72 |
| Eggs | Middle | 18 (40) | 0 (0-10) | 16 (34) | 0 (0-10) | .47 | 0.77 |
| Eggs | High | 15 (33) | 0 (0-0) | 18 (33) | 0 (0-17) | .12 | 0.78 |
| Nuts | Low | 9 (20) | 0 (0-0) | 10 (18) | 0 (0-15) | .65 | 0.88 |
| Nuts | Middle | 15 (31) | 0 (0-16) | 14 (34) | 0 (0-18) | .24 | 0.82 |
| Nuts | High | 17 (33) | 0 (0-20) | 17 (30) | 0 (0-20) | .86 | 0.85 |
| Milk (products) | Low | 339 (257) | 287 (175-500) | 363 (259) | 333 (180-519) | .40 | 0.68 |
| Milk (products) | Middle | 261 (279) | 216 (18-376) | 270 (222) | 221 (100-418) | .58 | 0.80 |
| Milk (products) | High | 238 (246) | 216 (0-349) | 275 (261) | 250 (32-392) | .01 | 0.85 |
| Cheese | Low | 28 (26) | 26 (0-48) | 36 (42) | 31 (0-45) | .23 | 0.70 |
| Cheese | Middle | 33 (37) | 29 (0-57) | 38 (39) | 31 (0-62) | .13 | 0.78 |
| Cheese | High | 35 (39) | 30 (0-58) | 42 (49) | 30 (0-68) | .048 | 0.76 |
| Bread | Low | 144 (117) | 135 (80-175) | 136 (77) | 140 (100-171) | .42 | 0.87 |
| Bread | Middle | 128 (90) | 120 (60-189) | 129 (82) | 132 (70-175) | .85 | 0.81 |
| Bread | High | 162 (127) | 132 (81-217) | 147 (97) | 120 (90-190) | .63 | 0.86 |
| Cereal products | Low | 44 (97) | 0 (0-26) | 61 (110) | 0 (0-61) | .07 | 0.83 |
| Cereal products | Middle | 52 (94) | 0 (0-60) | 64 (96) | 15 (0-100) | .22 | 0.76 |
| Cereal products | High | 89 (167) | 26 (0-112) | 87 (112) | 30 (0-155) | .05 | 0.83 |
| Potatoes | Low | 55 (81) | 0 (0-100) | 68 (103) | 0 (0-140) | .03 | 0.98 |
| Potatoes | Middle | 72 (124) | 0 (0-127) | 68 (116) | 0 (0-120) | .44 | 0.86 |
| Potatoes | High | 77 (127) | 0 (0-130) | 62 (93) | 0 (0-97) | .07 | 0.87 |
| Drinks | Low | 1,725 (942) | 1,620 (1,147-2,185) | 2,054 (931) | 1,943 (1,672-2,453) | .09 | 0.426 |
| Drinks | Middle | 1,751 (829) | 1,680 (1,300-2,140) | 1,977 (848) | 1,849 (1,427-2,288) | <.001 | 0.67 |
| Drinks | High | 2,073 (1,040) | 1,945 (1,400-2,656) | 2,218 (901) | 2,052 (1,617-2,735) | .03 | 0.78 |
| Sandwich spreads | Low | 16 (31) | 0 (0-20) | 16 (29) | 0 (0-19) | .63 | 0.91 |
| Sandwich spreads | Middle | 11 (26) | 0 (0-6) | 10 (25) | 0 (0-8) | .38 | 0.85 |
| Sandwich spreads | High | 18 (25) | 6 (0-26) | 13 (18) | 5 (0-22) | .005 | 0.89 |
| Snacks | Low | 99 (100) | 82 (23-123) | 98 (96) | 65 (21-146) | .89 | 0.84 |
| Snacks | Middle | 94 (144) | 55 (15-111) | 81 (88) | 55 (10-140) | .85 | 0.90 |
| Snacks | High | 85 (101) | 50 (12-113) | 78 (87) | 55 (15-98) | .33 | 0.85 |
| Sauces | Low | 31 (50) | 4 (0-44) | 43 (54) | 26 (0-62) | .02 | 0.64 |
| Sauces | Middle | 18 (32) | 1 (0-24) | 30 (36) | 12 (0-50) | .006 | 0.53 |
| Sauces | High | 20 (36) | 2 (0-25) | 31 (31) | 24 (0-58) | <.001 | 0.66 |
| Other | Low | 15 (36) | 0 (0-18) | 8 (13) | 0 (0-17) | .32 | 0.54 |
| Other | Middle | 12 (23) | 1 (0-14) | 5 (15) | 0 (0-6) | <.001 | 0.48 |
| Other | High | 14 (71) | 0 (0-7) | 3 (9) | 0 (0-0) | <.001 | 0.47 |

^a^Food groups are Wheel of Five food groups —main groups [23]. The food groups Fish, Legumes, and Soups were excluded because as the 75th percentile was 0 for both methods. See Table 3 provides more information on these food groups.

^b^The educational level concerned the participants’ highest completed educational level. Educational level was categorised into low (primary education, lower vocational education, advanced elementary education), middle (intermediate vocational education, higher secondary education) and high (higher vocational education and university).
^c^Wilcoxon signed rank test (normal approximation) of the differences between intake assessed using the DitEetIk! app and the GloboDiet 24-hour dietary recalls for the same day.
