# Supplementary material for: A Smartphone Food Record App Developed for the Dutch National Food Consumption Survey: Relative Validity Study
Source: JMIR Mhealth Uhealth. 2024 Feb 9;12:e50196. doi: 10.2196/50196 (PMC10891498; doi:10.2196/50196)
Supplement: Multimedia Appendix 2 [file mhealth_v12i1e50196_app2.docx]

Multimedia Appendix 2. The mean, median and IQR of consumption of food groups (grams per day) by BMI as assessed using the DitEetIk! app and 24-hour dietary recalls for the same day and their correlation for 211 participants with plausible energy intakes.

| Food group^a^ | BMI^b^ | DitEetIk! app food record (grams per day) | | GloboDiet 24-hour dietary recall (grams per day) | | Wilcoxon signed rank test P value^c^ | Spearman correlation coefficient |
| --- | --- | --- | --- | --- | --- | --- | --- |
|  |  | Mean (SD) | Median (IQR) | Mean (SD) | Median (IQR) |  |  |
|  |  |  |  |  |  |  |  |
| Vegetables | Normal | 162 (163) | 112 (44-255) | 147 (120) | 120 (50-218) | .74 | 0.74 |
| Vegetables | Overweight | 164 (260) | 117 (20-214) | 175 (159) | 158 (51-273) | .02 | 0.77 |
| Vegetables | Obesity | 163 (169) | 138 (24-205) | 162 (157) | 121 (52-240) | .61 | 0.76 |
| Fruit | Normal | 136 (167) | 97 (0-210) | 144 (160) | 130 (0-225) | .49 | 0.82 |
| Fruit | Overweight | 102 (125) | 62 (0-130) | 135 (145) | 128 (0-194) | .009 | 0.78 |
| Fruit | Obesity | 148 (260) | 83 (0-180) | 138 (127) | 134 (0-217) | .16 | 0.75 |
| Added fats | Normal | 16 (18) | 12 (1-24) | 20 (16) | 18 (8-31) | <.001 | 0.66 |
| Added fats | Overweight | 15 (14) | 12 (3-25) | 20 (14) | 21 (7-30) | .04 | 0.31 |
| Added fats | Obesity | 17 (15) | 15 (8-22) | 17 (16) | 14 (5-26) | .89 | 0.65 |
| Meat | Normal | 100 (100) | 72 (30-131) | 89 (81) | 75 (28-119) | .19 | 0.71 |
| Meat | Overweight | 109 (132) | 79 (16-143) | 97 (96) | 75 (30-125) | .24 | 0.74 |
| Meat | Obesity | 99 (105) | 72 (29-115) | 89 (71) | 77 (49-135) | .86 | 0.64 |
| Eggs | Normal | 14 (33) | 0 (0-0) | 17 (35) | 0 (0-8) | .12 | 0.85 |
| Eggs | Overweight | 21 (44) | 0 (0-13) | 19 (39) | 0 (0-17) | .97 | 0.75 |
| Eggs | Obesity | 15 (33) | 0 (0-0) | 14 (27) | 0 (0-15) | .92 | 0.65 |
| Nuts | Normal | 11 (23) | 0 (0-14) | 17 (32) | 0 (0-25) | .05 | 0.77 |
| Nuts | Overweight | 21 (40) | 0 (0-20) | 16 (33) | 0 (0-19) | .09 | 0.86 |
| Nuts | Obesity | 12 (26) | 0 (0-20) | 9 (20) | 0 (0-0) | .24 | 0.94 |
| Milk (products) | Normal | 231 (249) | 216 (10-342) | 279 (230) | 261 (73-423) | .001 | 0.83 |
| Milk (products) | Overweight | 282 (269) | 222 (66-415) | 297 (278) | 221 (99-424) | .47 | 0.89 |
| Milk (products) | Obesity | 292 (273) | 247 (46-420) | 293 (238) | 253 (100-402) | .71 | 0.64 |
| Cheese | Normal | 33 (36) | 30 (0-54) | 39 (44) | 31 (0-61) | .16 | 0.73 |
| Cheese | Overweight | 31 (36) | 24 (0-55) | 40 (46) | 31 (0-62) | .05 | 0.74 |
| Cheese | Obesity | 36 (37) | 31 (0-60) | 39 (42) | 31 (0-62) | .15 | 0.83 |
| Bread | Normal | 167 (131) | 140 (70-220) | 148 (101) | 134 (80-210) | .44 | 0.83 |
| Bread | Overweight | 122 (89) | 115 (70-170) | 119 (72) | 120 (70-171) | .92 | 0.84 |
| Bread | Obesity | 143 (105) | 113 (70-195) | 147 (84) | 140 (80-190) | .17 | 0.87 |
| Cereal products | Normal | 69 (127) | 6 (0-93) | 68 (100) | 20 (0-104) | .51 | 0.84 |
| Cereal products | Overweight | 55 (92) | 19 (0-63) | 84 (115) | 30 (0-145) | .003 | 0.73 |
| Cereal products | Obesity | 78 (177) | 0 (0-108) | 70 (104) | 10 (0-119) | .49 | 0.82 |
| Potatoes | Normal | 72 (116) | 0 (0-127) | 63 (92) | 0 (0-97) | .87 | 0.87 |
| Potatoes | Overweight | 88 (143) | 0 (0-148) | 72 (123) | 0 (0-92) | .12 | 0.86 |
| Potatoes | Obesity | 50 (86) | 0 (0-98) | 62 (97) | 0 (0-143) | .01 | 0.93 |
| Drinks | Normal | 2,025 (993) | 1,920 (1,413-2,525) | 2,210 (941) | 2,000 (1,662-2,551) | .08 | 0.64 |
| Drinks | Overweight | 1,820 (761) | 1,830 (1,377-2,205) | 1,959 (701) | 1,963 (1,620-2,397) | .01 | 0.73 |
| Drinks | Obesity | 1,767 (1,095) | 1,575 (1,035-2,075) | 2,095 (1,000) | 1,857 (1,367-2,651) | <.001 | 0.66 |
| Sandwich spreads | Normal | 16 (26) | 0 (0-20) | 15 (25) | 5 (0-20) | .45 | 0.89 |
| Sandwich spreads | Overweight | 14 (26) | 0 (0-20) | 11 (24) | 0 (0-13) | .07 | 0.92 |
| Sandwich spreads | Obesity | 14 (29) | 0 (0-20) | 10 (20) | 0 (0-5) | .21 | 0.82 |
| Snacks | Normal | 97 (102) | 61 (29-131) | 96 (99) | 63 (27-140) | .37 | 0.89 |
| Snacks | Overweight | 75 (109) | 36 (1-100) | 68 (83) | 38 (2-100) | .96 | 0.94 |
| Snacks | Obesity | 100 (151) | 50 (26-115) | 80 (78) | 55 (25-120) | .59 | 0.78 |
| Sauces | Normal | 25 (41) | 5 (0-32) | 31 (34) | 22 (0-51) | .04 | 0.64 |
| Sauces | Overweight | 24 (42) | 0 (0-32) | 33 (44) | 20 (0-47) | .02 | 0.58 |
| Sauces | Obesity | 13 (21) | 1 (0-21) | 35 (36) | 24 (0-62) | <.001 | 0.59 |
| Other | Normal | 14 (27) | 3 (0-18) | 7 (16) | 0 (0-9) | <.001 | 0.68 |
| Other | Overweight | 9 (22) | 0 (0-5) | 4 (8) | 0 (0-3) | .02 | 0.35 |
| Other | Obesity | 16 (91) | 0 (0-3) | 3 (9) | 0 (0-0) | .009 | 0.28 |

^a^Food groups are Wheel of Five food groups —main groups [23]. The food groups Fish, Legumes, and Soups were excluded because as the 75th percentile was 0 for both methods. See Table 3 provides more information on these food groups.

^b^ BMI cut-offs for adults: Normal weight 18.5 to <25; Overweight 25 to <30; Obesity ≥ 30 kg/m2.
^c^Wilcoxon signed rank test (normal approximation) of the differences between intake assessed using the DitEetIk! app and the GloboDiet 24-hour dietary recalls for the same day.
